# Multiple mutations of SARS-CoV-2 Omicron BA.2 variant orchestrate its virological characteristics

**DOI:** 10.1128/jvi.01011-23

**Published:** 2023-10-05

**Authors:** Izumi Kimura, Daichi Yamasoba, Hesham Nasser, Hayato Ito, Jiri Zahradnik, Jiaqi Wu, Shigeru Fujita, Keiya Uriu, Jiei Sasaki, Tomokazu Tamura, Rigel Suzuki, Sayaka Deguchi, Arnon Plianchaisuk, Kumiko Yoshimatsu, Yasuhiro Kazuma, Shuya Mitoma, Gideon Schreiber, Hiroyuki Asakura, Mami Nagashima, Kenji Sadamasu, Kazuhisa Yoshimura, Akifumi Takaori-Kondo, The Genotype to Phenotype Japan (G2P-Japan) Consortium, Naoko Misawa, Jumpei Ito, Kotaro Shirakawa, Kazuo Takayama, Takashi Irie, Takao Hashiguchi, So Nakagawa, Takasuke Fukuhara, Akatsuki Saito, Terumasa Ikeda, Kei Sato

**Affiliations:** 1 Institute of Medical Science, University of Tokyo, Tokyo, Japan; 2 Joint Research Center for Human Retrovirus infection, Kumamoto University, Kumamoto, Japan; 3 Hokkaido University, Sapporo, Japan; 4 Kyoto University, Kyoto, Japan; 5 University of Miyazaki, Miyazaki, Japan; 6 Tokyo Metropolitan Institute of Public Health, Tokyo, Japan; 7 Hiroshima University, Hiroshima, Japan; 8 One Health Research Center, Hokkaido University, Sapporo, Japan; 9 International Institute for Zoonosis Control, Hokkaido University, Sapporo, Japan; 10 Hokkaido University, Sapporo, Japan; 11 Joint Research Center for Human Retrovirus infection, Kumamoto, Japan; 12 Kyushu University, Fukuoka, Japan; 1 Division of Systems Virology, Department of Microbiology and Immunology, The Institute of Medical Science, The University of Tokyo, Tokyo, Japan; 2 Faculty of Medicine, Kobe University, Kobe, Japan; 3 Division of Molecular Virology and Genetics, Joint Research Center for Human Retrovirus infection, Kumamoto University, Kumamoto, Japan; 4 Department of Clinical Pathology, Faculty of Medicine, Suez Canal University, Ismailia, Egypt; 5 Department of Microbiology and Immunology, Faculty of Medicine, Hokkaido University, Sapporo, Japan; 6 Department of Biomolecular Sciences, Weizmann Institute of Science, Rehovot, Israel; 7 First Medical Faculty at Biocev, Charles University, Vestec-Prague, Czechia; 8 Department of Molecular Life Science, Tokai University School of Medicine, Isehara, Japan; 9 Graduate School of Medicine, The University of Tokyo, Tokyo, Japan; 10 Laboratory of Medical Virology, Institute for Life and Medical Sciences, Kyoto University, Kyoto, Japan; 11 Institute for Vaccine Research and Development (HU-IVReD), Hokkaido University, Sapporo, Japan; 12 Center for iPS Cell Research and Application (CiRA), Kyoto University, Kyoto, Japan; 13 Institute for Genetic Medicine, Hokkaido University, Sapporo, Japan; 14 Department of Hematology and Oncology, Graduate School of Medicine, Kyoto University, Kyoto, Japan; 15 Department of Veterinary Science, Faculty of Agriculture, University of Miyazaki, Miyazaki, Japan; 16 Graduate School of Medicine and Veterinary Medicine, University of Miyazaki, Miyazaki, Japan; 17 Tokyo Metropolitan Institute of Public Health, Tokyo, Japan; 18 International Research Center for Infectious Diseases, The Institute of Medical Science, The University of Tokyo, Tokyo, Japan; 19 AMED-CREST, Japan Agency for Medical Research and Development (AMED), Tokyo, Japan; 20 Graduate School of Biomedical and Health Sciences, Hiroshima University, Hiroshima, Japan; 21 CREST, Japan Science and Technology Agency, Saitama, Japan; 22 Bioinformation and DDBJ Center, National Institute of Genetics, Mishima, Japan; 23 Laboratory of Virus Control, Research Institute for Microbial Diseases, Osaka University, Suita, Japan; 24 Center for Animal Disease Control, University of Miyazaki, Miyazaki, Japan; 25 International Vaccine Design Center, The Institute of Medical Science, The University of Tokyo, Tokyo, Japan; 26 Graduate School of Frontier Sciences, The University of Tokyo, Kashiwa, Japan; 27 Collaboration Unit for Infection, Joint Research Center for Human Retrovirus infection, Kumamoto University, Kumamoto, Japan; University of North Carolina at Chapel Hill, Chapel Hill, North Carolina, USA

**Keywords:** SARS-CoV-2, COVID-19, Omicron, BA.1, BA.2, immune resistance, growth capacity, fusogenicity, pathogenicity

## Abstract

Previous studies on the Omicron BA.2 variant suggested that the virological characteristics of BA.2 are determined by the mutations in at least two different regions of the viral genome: in the BA.2 *spike* gene (enhancing viral fusogenicity and intrinsic pathogenicity) and the non-*spike* region of the BA.2 genome (leading to intrinsic pathogenicity attenuation). However, the mutations modulating the BA.2 virological properties remain elusive. In this study, we demonstrated that the L371F substitution in the BA.2 spike protein confers greater fusogenicity and intrinsic pathogenicity. Furthermore, we revealed that multiple mutations downstream of the *spike* gene in the BA.2 genome are responsible for attenuating intrinsic viral pathogenicity and replication capacity. As mutations in the SARS-CoV-2 variant spike proteins could modulate certain virological properties, such as immune evasion and infectivity, most studies have previously focused on spike protein mutations. Our results underpin the importance of non-spike protein-related mutations in SARS-CoV-2 variants.

**Importance:**

Most studies investigating the characteristics of emerging SARS-CoV-2 variants have been focusing on mutations in the spike proteins that affect viral infectivity, fusogenicity, and pathogenicity. However, few studies have addressed how naturally occurring mutations in the non-*spike* regions of the SARS-CoV-2 genome impact virological properties. In this study, we proved that multiple SARS-CoV-2 Omicron BA.2 mutations, one in the spike protein and another downstream of the *spike* gene, orchestrally characterize this variant, shedding light on the importance of Omicron BA.2 mutations out of the spike protein.

## INTRODUCTION

Until the end of 2021, SARS-CoV-2 has diversified, and several variants of concern (VOCs), such as Alpha, Beta, Gamma, and Delta, emerged and spread worldwide. In November 2021, Omicron BA.1, a novel VOC, was detected in South Africa, rapidly spread globally, and outcompeted prior VOCs. Soon after this global spread, BA.2, another Omicron lineage emerged at the beginning of 2022 and outcompeted BA.1. Although several VOCs (such as the BA.5, BA.2.75, BQ.1, and XBB lineages) have emerged after the global spread of BA.2, all these variants derived from BA.2, indicating the global predominance of BA.2 descendants over a single year (1).

Higher transmissibility (2 – 4), profound resistance against vaccination- and natural infection-induced antiviral humoral immunity (2, 3), reduced viral spike (S) protein fusogenicity (2, 3, 5), and attenuated pathogenicity in experimentally infected hamsters (here, we refer to it as “intrinsic pathogenicity”) (2, 3) commonly characterize early Omicron subvariants including BA.1 and BA.2, mainly determined by the viral S protein. In our recent study, we demonstrated that the S:S375F substitution, common both in BA.1 and BA.2, mainly contributes to the Omicron BA.1 virological characteristics such as reduced fusogenicity (6). Intriguingly, our phylogenetic analysis suggested that the acquisition of the S:S375F substitution is associated with triggering an explosive BA.1 spread (6).

As mentioned above, the S:S375F substitution is common both in BA.1 and BA.2, and it could thus be a critical mutation that characterizes the virological features of Omicron. However, BA.2 outcompeted BA.1, suggesting the virological superiority and difference of BA.2 compared to BA.1. For instance, the BA.2 S receptor-binding domain (RBD) exhibits higher affinity to the human ACE2 receptor than that of the BA.1 S protein (3, 7, 8). BA.2 is more resistant to Sotrovimab, a therapeutic monoclonal antibody, than BA.1 and the ancestral strain (3, 9, 10). More importantly, the BA.2 S protein is more fusogenic than that of BA.1, and the intrinsic pathogenicity of a BA.2 S-carrying recombinant virus is higher than that of a BA.1 S-carrying recombinant virus in hamsters (3). In sharp contrast, the intrinsic pathogenicity of BA.2 clinical isolates is comparable to that of BA.1 clinical isolates in hamsters (11, 12). These observations suggest that the BA.2 virological features could be determined by mutations in at least two different regions of the viral genome: one in the BA.2 S protein could increase viral fusogenicity and intrinsic pathogenicity and another in the non-*S* region of the BA.2 genome could attenuate intrinsic pathogenicity in hamsters. However, the mutations modulating BA.2 virological characteristics remain elusive. In this study, our molecular phylogenetic analyses suggest that the BA.2 *S* gene was derived from the BA.1 *S* gene. Our *in vitro* cell culture and *in vivo* hamster model-related experiments showed that the S:L371F substitution determines enhanced fusogenicity, increased RBD protein stability, and higher intrinsic pathogenicity. Moreover, our data indicate that multiple mutations downstream of the *S* gene in the BA.2 genome are responsible for attenuating intrinsic viral pathogenicity and replication capacity.

## RESULTS

### BA.2 *S* evolution from BA.1 *S*

**Fig 1:**
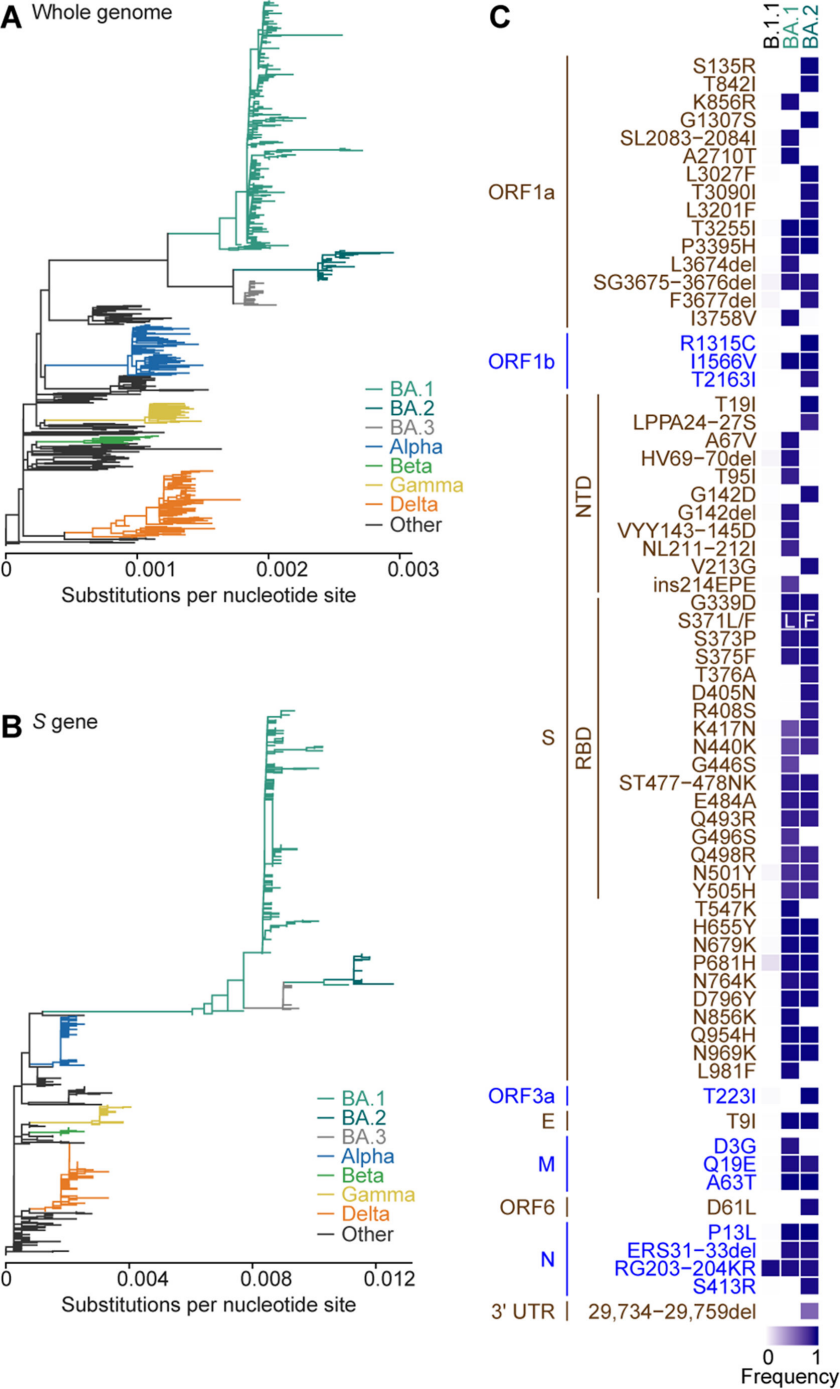
Evolution of BA.1 and BA.2 lineages. (**A and B**) The ML trees of 624 SARS-CoV-2 genomes (**A**) and *S* gene region (**B**). Each branch color corresponds to the SARS-CoV-2 PANGO lineage shown on the right. The scale (substitutions per nucleotide site) is shown at the bottom. The trees with mutations and bootstrap values of the trees are shown in Fig. S1. (**C**) A heatmap showing frequency of mutation occurred in proteins and 3′UTR of SARS-CoV-2 in BA.1 and BA.2 lineages compared to those in B.1.1 lineage.

To investigate the evolution of the SARS-CoV-2 Omicron *S* gene, we performed the molecular phylogenetic analysis of 624 SARS-CoV-2 genomes, including 349 Omicron genomes (see Materials and Methods). Consistent with previous studies (13, 14), the maximum likelihood (ML)-based phylogenetic tree of the SARS-CoV-2 genome sequences revealed that the Omicron strain comprises three major sublineages: BA.1, BA.2, and BA.3, forming distinct clusters in the phylogenetic tree (Fig. 1A; a tree including mutations and bootstrap values are shown in Fig. S1A). However, the phylogenetic tree of the *S*-encoding region exhibited a different topology (Fig. 1B; a tree including mutations and bootstrap values are shown in Fig. S1B) and the BA.2/3 lineages were nested in the BA.1 clade. Although the bootstrap value of *S* in the root of the BA.2 lineage was not high (51%) (Fig. S1B), the evolutionary relationship between BA.1 and BA.2/3 in the *S* gene was further supported by the phylogenetic trees of the RBD-encoding region of the *S* gene (Fig. S1C). These findings suggest that the *S* gene, including the RBD-encoding region, of BA.2 and BA.3 could have been derived from BA.1.

### S:L371F substitution impacts the BA.2 S virological features

**Fig 2:**
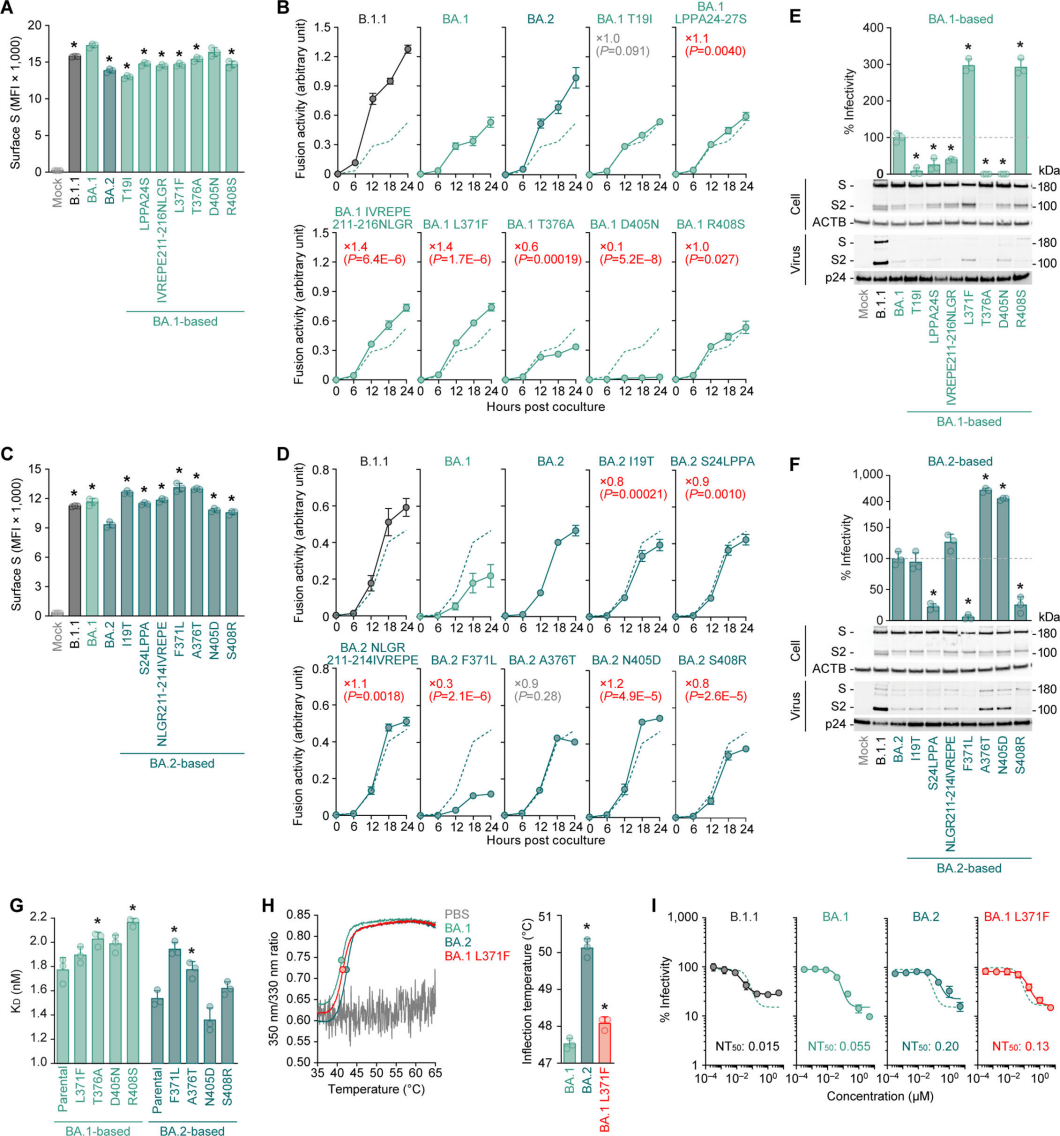
Impact of L371F substitution on the functions of Omicron S. (A–D) S-based fusion assay. Cell surface expression of BA.1-based derivatives (**A**) and BA.2-based derivatives (**C**) is shown. (**B and D**) S-based fusion assay in Calu-3 cells. The recorded fusion activity (arbitrary units) is shown. The dashed green lines in panels (**B**) and (**D**) are the results of BA.1 S and BA.2 S, respectively. The red number in each panel indicates the fold difference between BA.1 (top) or BA.2 (bottom) and the derivative tested at 24 h post coculture. (**E and F**) Pseudovirus assay and western blotting. Top, HOS-ACE2-TMPRSS2 cells were infected with pseudoviruses bearing each S protein. The amount of input virus was normalized based on the amount of HIV-1 p24 capsid protein. The percent infectivity compared to that of the virus pseudotyped with BA.1 S (**E**) or BA.2 S (**F**) is, respectively, shown. The direct comparison between BA.1 S and BA.2 is shown in Fig. S2. The dashed horizontal lines in the left and right panels indicate the values of BA.1 and BA.2, respectively. Bottom, western blot. Representative blots of S-expressing cells (labeled with “Cell”) and supernatants (labeled with “Virus”) are shown. ACTB and HIV-1 p24 were used for the internal controls of “Cell” and “‘Virus.” kDa, kilodalton. In Fig. 2A through F, the three mutations in the NTD, NL211-212I, V213G, and ins214EPE (shown in Fig. 1C) are combined to either IVREPE211-216NLGR (for BA.1 S) or NLGR211-214IVREPE (for BA.2 S). (**G**) The binding affinity of the RBD of SARS-CoV-2 S protein to ACE2 by yeast surface display. The *K*_*D*_ value indicating the binding affinity of the RBD of the SARS-CoV-2 S protein to soluble ACE2 when expressed in yeast is shown. (**H**) DSF assay. Representative results (left) and the summarized data of the inflection temperatures of the RBD proteins by BA.1, BA.1 L371F, or BA.2 (right) are shown. (**I**) Neutralization assay using Sotrovimab. NT_50_, 50% neutralization titer. The dashed green lines are the results of BA.1. Assays were performed in triplicate. The presented data are expressed as the average ± SD. In panels (**A**), (**C**), (**E**), (**F**), (**G**), and (H, right), each dot indicates the result of an individual replicate. Statistically significant differences (*P < 0.05) versus parental BA.1 [panels (**A**), (**E**), (G, left), and (H, right)] or parental BA.2 [(**C**), (**F**),and (G, right)] were determined by two-sided Student’s *t*-tests. In panels (**B**) and (**D**), statistically significant differences versus parental BA.1 (**B**) or parental BA.2 (**D**) across timepoints were determined by multiple regression. The FWERs calculated using the Holm method are indicated with parentheses in the figures.

As mentioned in the Introduction, previous studies suggested that the viral features of BA.2 could be determined by mutations in at least two different genomic regions: one in the BA.2 S protein increases viral fusogenicity and intrinsic pathogenicity and other in the non-*S* region attenuates viral pathogenicity (3, 11). Compared to the BA.1 S protein, the BA.2 S protein harbors seven unique mutations: T19I, LPPA24-27S, IVREPE211-216NLGR (including V213G), L371F, T376A, D405N, and R408S [Fig. 1C; note that G142D is not specific for BA.2 as this substitution was already detected in the Delta variant (15)]. To determine the mutations that increase viral fusogenicity, we prepared plasmids expressing the S proteins of BA.1, BA.2, and a series of BA.1/2 derivatives carrying single substitutions. In the case of BA.1 S derivatives, substitutions other than D405N slightly reduced the cell surface expression levels of the S protein compared to that of the parental BA.1 S protein (Fig. 2A). The S-based fusion assay (16) demonstrated that LPPA24-27S, IVREPE211-216NLGR, and L371F mutations were significantly more fusogenic than parental BA.1 S protein (1.1-, 1.4-, and 1.4-fold, respectively) (Fig. 2B). Unexpectedly, D405N severely attenuated BA.1 S-mediated fusogenicity (Fig. 2B). In the case of BA.2 S derivatives, all substitutions significantly increased the cell surface expression levels of the S protein (Fig. 2C). More importantly, I19T, S24LPPA, F371L, and S408R severely reduced fusogenicity (by 0.8-, 0.9-, 0.3-, and 0.8-fold, respectively) (Fig. 2D).

Next, we used the expression plasmids of BA.1 and BA.2 S derivatives for preparing HIV-1-based pseudoviruses and measured their infectivity. Consistent with our previous report (3), BA.2 pseudoviruses displayed significantly higher infectivity (3.8-fold) than BA.1 pseudoviruses (Fig. S2).

L371F and R408S significantly increased the BA.1 S-based pseudovirus infectivity (by 3.0- and 2.9-fold, respectively) (Fig. 2E, top). However, S24LPPA, F371L, and S408R significantly reduced the BA.2 S-based pseudovirus infectivity (by 0.2-, 0.1-, and 0.3-fold, respectively) (Fig. 2F, top). Western blotting of pseudovirus-producing cells demonstrated that the S protein expression levels were comparable to those of the parental S (Fig. 2E and F). Therefore, not the protein expression level—but the trafficking efficacy-related differences caused the different surface expression levels of the S proteins (Fig. 2A and C). Moreover, our western blotting analysis of the pseudoviruses in the culture supernatant revealed that the virion-incorporated S2 protein level was different in the case of certain S derivatives (Fig. 2E and F). For example, the S2 protein levels of L371F and D405N in the case of BA.1 pseudoviruses were higher than that of the parental BA.1 virus (Fig. 2E), and those of A376T and N405D in the case of BA.2 pseudoviruses were higher than that of the parental BA.2 virus (Fig. 2F). These results suggest that the S protein mutations modulate the S2 protein incorporation or cleavage efficiency into released pseudoviruses.

Four of the seven mutations in the BA.2 S protein compared to the BA.1 S protein were located in the RBD: L371F, T376A, D405N, and R408S. As L371F is in the RBD, we hypothesized that the L371F-related increased fusogenicity and pseudovirus infectivity could be attributed to increased ACE2-binding affinity. To address this possibility, we prepared expression plasmids of the BA.1 or BA.2 S RBD derivatives. Consistent with our previous study (3), our yeast surface display assay (8, 17) demonstrated that the BA.2 S RBD *K*_*D*_ value was significantly lower than that of the BA.1 S RBD (Fig. 2G), suggesting higher ACE2-binding affinity in the case of BA.2 S RBD compared to that of the BA.1 S RBD. However, the BA.1 S RBD derivatives containing respective substitutions in the BA.2 S RBD showed that none of these four substitutions increased the S RBD ACE2-binding affinity (Fig. 2G, left). The T376A and R408S substitutions significantly increased the *K*_*D*_ values instead (Fig. 2G, left). However, the two BA.2 S RBD derivatives carrying F371L and A376T exhibited significantly higher *K*_*D*_ values compared to the parental BA.2 S RBD, BA.2 F371L displaying the highest value among the BA.2 S RBD-based mutants (Fig. 2G, right).

The results of the cell-based fusion assay (Fig. 2A through D), pseudovirus assay (Fig. 2E and F), and yeast surface display (Fig. 2G) presented a certain level of inconsistency with each other. In the pseudovirus assay (Fig. 2E and F), both substitutions of residues 371 and 408 were reciprocal: the L371F and R408S substitutions significantly increased the BA.1 pseudovirus infectivity, while F371L and S408R significantly reduced that of the BA.2 pseudovirus. These data suggested that these two substitutions are important to characterize the BA.2 viral features. However, the cell-based fusion assay revealed that the substitution at residue 408 did not critically affect the fusogenicity of the BA.1 and BA.2 S proteins (Fig. 2B and D). On the other hand, consistent with the pseudovirus assay results (Fig. 2E and F), those of the cell-based fusion assay of the substitution at residue 371 were reciprocal: BA.1 L371F significantly increased (Fig. 2B), while BA.2 F371L significantly reduced fusogenicity (by 1.4- and 0.3-fold, Fig. 2B and D, respectively). As we have previously reported that S protein fusogenicity is closely associated with viral pathogenicity (2, 3, 14, 15), we could reasonably assume that L371F is a key mutation for the higher pathogenicity in the case of BA.2 S protein compared to the BA.1 S protein. Based on the structure, hydrophobic and aromatic amino acid residues accumulated around RBD L371F, suggesting the contribution of this site to RBD folding. To address this question, we performed a differential scanning fluorimetry (DSF) assay using the RBD proteins of BA.1, BA1-based L371F, and BA.2 (Fig. 2H). We observed that the BA.2 S RBD thermostability was significantly higher than that of BA.1 S RBD (Fig. 2H). Importantly, BA.1 S thermostability significantly increased upon the L371F substitution (Fig. 2H). In summary, these results suggest that the increased BA.2 S fusogenicity could be partly explained by the L371F substitution, and the improved BA.2 S fusogenicity is likely attributed to the increased RBD-folding stability compared to the BA.1 S protein.

### S:L371F substitution is responsible for the Sotrovimab resistance

Previous studies, including ours, demonstrated that BA.2 is resistant while BA.1 is sensitive to Sotrovimab, a therapeutic monoclonal antibody (3, 18 – 20). To assess the potential association of the L371F substitution to the Sotrovimab resistance, we performed a neutralization assay. Both the BA.1 S:L371F and BA.2 were Sotrovimab-resistant, suggesting that the S:L371F substitution partly contributes to such resistance (Fig. 2I).

### S:L371F substitution impacts viral growth, fusogenicity, and intrinsic pathogenicity

**Fig 3:**
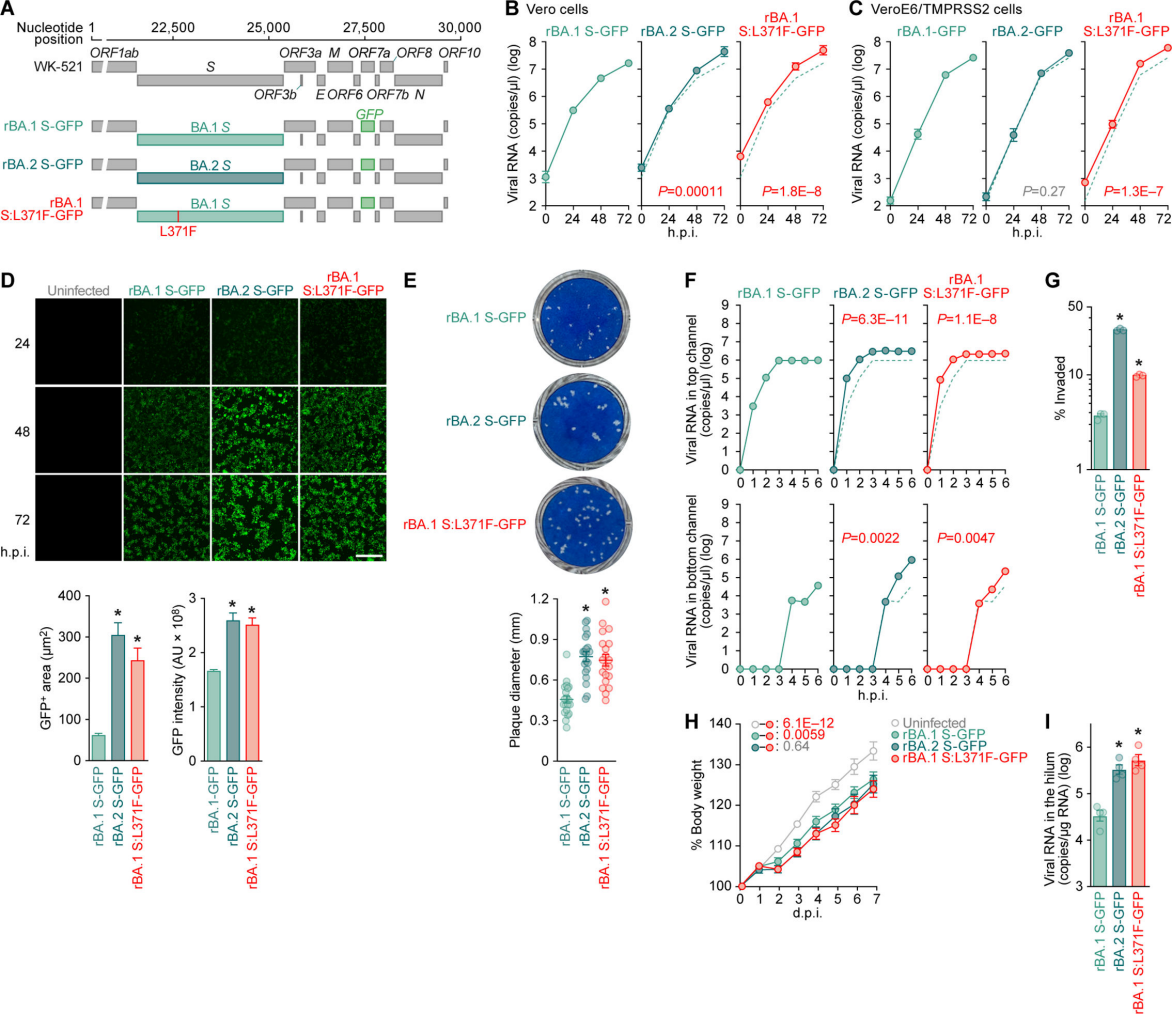
Impact of S:L371F substitution on the virological features of Omicron. (**A**) Scheme for the chimeric recombinant SARS-CoV-2 used in this study. The SARS-CoV-2 genome and its genes are shown. The template was SARS-CoV-2 strain WK-521 (PANGO lineage A, GISAID ID: EPI_ISL_408667) (22), and *ORF7a* gene was swapped with the *GFP* gene. Two recombinant viruses, rBA.1 S-GFP and rBA.2 S-GFP, were used in our previous study (3). (**B and C**) Viral growth assay. rBA.1 S-GFP, rBA.2 S-GFP, and rBA.1 S:L371F-GFP were inoculated into Vero cells [B; multiplicity of infection (m.o.i.) = 0.1] and VeroE6/TMPRSS2 cells (C; m.o.i. = 0.01). The copy numbers of viral RNA in the culture supernatant were routinely quantified by RT–qPCR. (**D**) Fluorescence microscopy. The GFP area was measured in infected VeroE6/TMPRSS2 cells (m.o.i. = 0.01) at 48 h.p.i. Representative panels are shown in the left panel. Scale bars, 400 μm. Middle and right, the summarized results of GFP-positive area (middle) and GFP intensity (right). To measure the GFP-positive area, 1,000 cells per virus were counted. (**E**) Plaque assay. Representative panels (left) and a summary of the recorded plaque diameters (20 plaques per virus) (right) are shown. (**F and G**) Viral growth in an airway-on-a-chip system. rBA.1 S-GFP, rBA.2 S-GFP, and rBA.1 S:L371F-GFP were inoculated into an airway-on-a-chip system, and the copy numbers of viral RNA in the top (F, top) and bottom (F, bottom) channels of an airway-on-a-chip were routinely quantified by RT–qPCR. (**G**) The percentage of viral RNA load in the bottom channel per top channel during 6 d.p.i. (i.e., percentage of invaded virus from the top channel to the bottom channel) is shown. (**H and I**) Animal experiment. Syrian hamsters (*n* = 6 per group) were intranasally inoculated with rBA.1 S-GFP, rBA.2 S-GFP, and rBA.1 S:L371F-GFP (10,000 TCID_50_ in 100 µL per animal). Hamsters of the same age were intranasally inoculated with 100 µL of saline (uninfected). (**H**) Body weight change of infected hamsters (*n* = 6 per infection group). (**I**) Viral RNA loads in the lung hilum of infected hamsters at 5 d.p.i. (*n* = 4 per infection group). Assays were performed in triplicate [(**F**) and (**G**)] or quadruplicate [(**B**) and (**C**)]. The presented data are expressed as the average ± SEM. In panels (**E**) and (**G**), each dot indicates the result of an individual replicate. In panel (**I**), each dot indicates the result of an individual hamster. In panels (**B**), (**C**), and (**F**), the dashed green lines are the results of rBA.1 S-GFP. Statistically significant differences (*P < 0.05) versus rBA.1 S-GFP were determined by two-sided Mann-Whitney *U* test [(**D**), (**E**), and (**I**)] or two-sided Student’s *t*-test (**G**). In panels (**B**), (**C**), (**F**), and (**H**), statistically significant differences versus rBA.1 S:L371F-GFP across timepoints were determined by multiple regression. The FWERs calculated using the Holm method are indicated in the figures.

To investigate how the S:L371F substitution could affect viral growth capacity, we artificially generated recombinant SARS-CoV-2 using the circular polymerase extension reaction (CPER) technique (21). The WK-521 strain (PANGO lineage A, GISAID ID: EPI_ISL_408667) (22) served as a backbone for the recombinant viruses and the *ORF7a* gene was replaced with the *green fluorescent protein* (*GFP*) gene (21) (Fig. 3A). As previously described (3), the *S* gene was swapped either with that from BA.1 (rBA.1 S-GFP) or BA.2 (rBA.2 S-GFP) (Fig. 3A). Additionally, we prepared a recombinant virus carrying the BA.1 S:L371F (rBA.1 S:L371F-GFP) (Fig. 3A). To generate a BA.2 S:F371L-carrying recombinant virus, we independently performed the CPER four times. However, this latter recombinant virus could not be rescued. CPER-mediated recombinant virus generation depends on the seed virus amplification via viral growth in the transfected cell culture (21), and we demonstrated that the BA.2 S:F371L substitution severely attenuated pseudoviral infectivity (Fig. 2F) and fusogenicity (Fig. 2D). Therefore, the failure to generate the recombinant S:F371L mutation-carrying BA.2 strongly suggests the importance of F371 in the BA.2 S-carrying viral infectivity.

Consistent with our previous study (3), rBA.2 S-GFP growth was more efficient than that of rBA.1 S-GFP in Vero (Fig. 3B) but not in VeroE6/TMPRSS2 cells (Fig. 3C). Interestingly, rBA.1 S:L371F-GFP growth was significantly more efficient than that of rBA.1 S-GFP in both cell lines (Fig. 3B and C). Notably, the GFP intensity expressed according to viral replication and the GFP-positive areas of both rBA.2 S-GFP and rBA.1 S:L371F-GFP were significantly higher than those of rBA.1 S-GFP (Fig. 3D). These results suggest that rBA.2 S-GFP forms significantly more extended syncytia than rBA.1 S-GFP, attributed to the S:L371F substitution. Moreover, the plaque sizes of rBA.2 S-GFP- and rBA.1 S:L371F-GFP-infected VeroE6/TMPRSS2 cells were significantly larger than those of rBA.1 S-GFP-infected VeroE6/TMPRSS2 cells (Fig. 3E). Furthermore, to measure how the S:L371F substitution affected the airway epithelial and endothelial barriers, we used an airway-on-a-chip system. By measuring the viral load invading the vascular channel from the airway channel, the airway epithelial and endothelial barrier-disrupting viral properties could be evaluated (12, 23, 24). We quantified the viral load in the airway and vascular channels (Fig. 3F, top and bottom, respectively) and observed that the percentage of viruses invading the vascular channel of the rBA.2 S-GFP- and rBA.1 S:L371F-GFP-infected airway-on-a-chips was significantly higher than that of the rBA.1 S-GFP-infected equivalents (Fig. 3G). Taken together, these results suggest that the L371F substitution partly contributes to the higher BA.2 S fusogenicity compared to BA.1 S protein.

Our previous studies on Delta (15), Omicron BA.1 (2), BA.2 (3), BA.5 (14), and BA.2.75 (24) suggested a close association between viral fusogenicity and intrinsic viral pathogenicity. As our data demonstrated that the L371F substitution increases BA.1 S fusogenicity (Fig. 2B and 3D through G), we reasonably assumed that the L371F substitution is associated with the increased BA.2 S-carrying viral pathogenicity we previously observed (3). To address this possibility, we intranasally inoculated rBA.1 S-GFP, rBA.2 S-GFP, and rBA.1 S:L371F-GFP into hamsters. Consistent with our previous study (3), the body weight of rBA.2 S-GFP-infected hamsters was significantly reduced compared to that of rBA.1 S-GFP-infected hamsters (Fig. 3H), suggesting that the BA.2 S-carrying virus is more pathogenic than the BA.1 S-carrying one. Moreover, the viral RNA load in the lung hilum of rBA.1 S:L371F GFP-infected hamsters 5 days postinfection (d.p.i.) was significantly higher than that of parental rBA.1 S-GFP and comparable to that of rBA.2 GFP-infected hamsters (Fig. 3I). These results suggest that the S:L371F substitution is closely associated with the increased pathogenicity of the BA.2 S-carrying virus compared to the BA.1 S-carrying virus.

### Multiple mutations downstream of the *S* gene synergically act to attenuate viral growth and intrinsic pathogenicity

**Fig 4:**
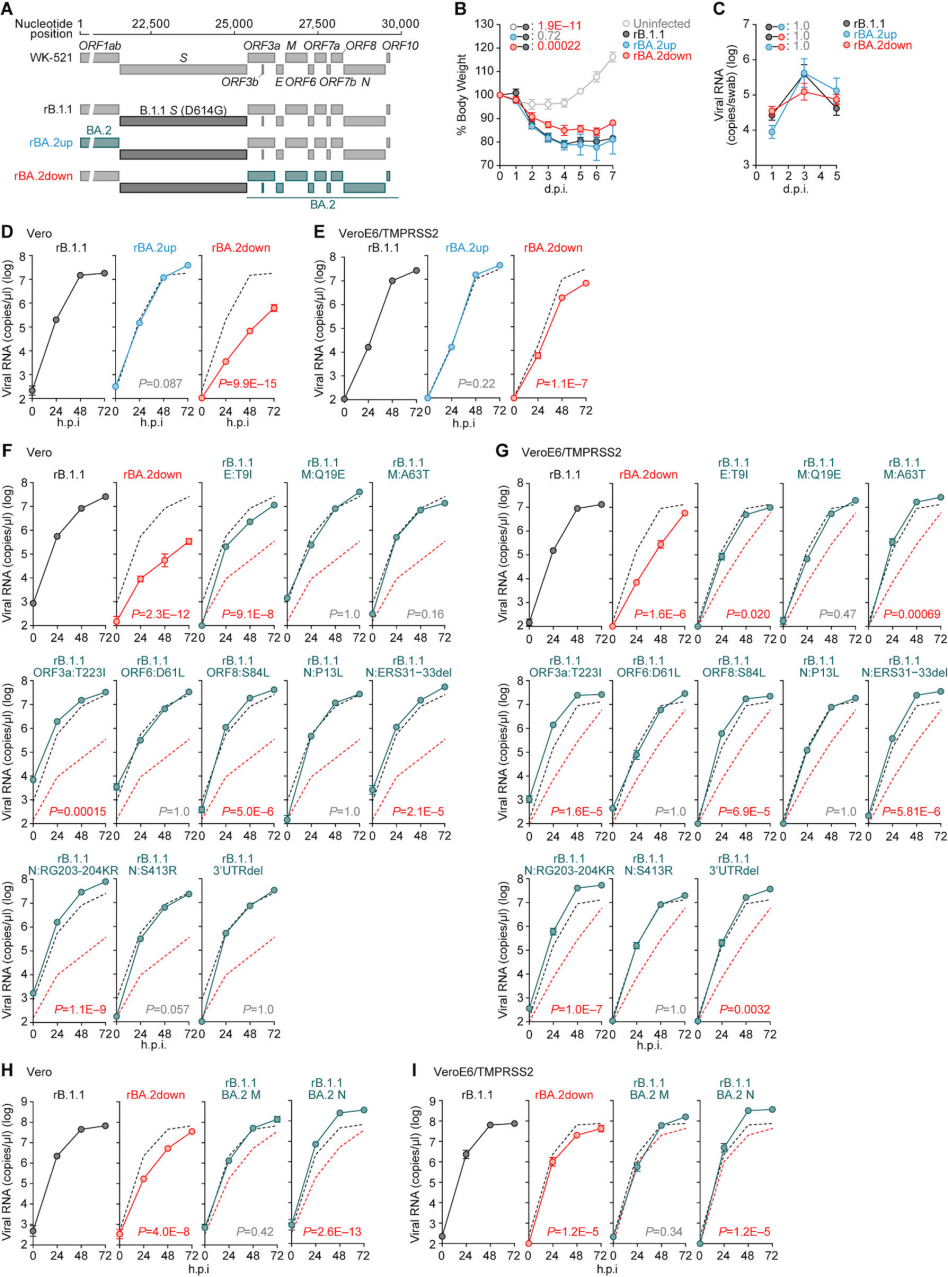
Modulation of viral growth and pathogenicity by the mutations downstream of *S* gene. (**A**) Scheme for the chimeric recombinant SARS-CoV-2 used in this study. The SARS-CoV-2 genome and its genes are shown. The template was SARS-CoV-2 strain WK-521 (PANGO lineage A, GISAID ID: EPI_ISL_408667) (22). A recombinant virus bearing S:D614G mutation (rB.1.1) was used in our previous study (3). (**B and C**) Animal experiment. Syrian hamsters (*n* = 4 per group) were intranasally inoculated with rB.1.1, rBA.2up, and rBA.2down (10,000 TCID_50_ in 100 µL per animal). Hamsters of the same age were intranasally inoculated with 100 µL of saline (uninfected). (**B**) Body weight change of infected hamsters. (**C**) Viral RNA loads in the oral swabs of infected hamsters at 1, 3, and 5 d.p.i. (D–I) Viral growth assay. rB.1.1 (black), rBA.2down (red), rBA.2up (blue), or the rB.1.1 derivatives bearing the mutation indicated in the figure were inoculated into Vero cells (D, F, and H; m.o.i. = 0.1) and VeroE6/TMPRSS2 cells (E, G, and I; m.o.i. = 0.01). The copy numbers of viral RNA in the culture supernatant were routinely quantified by RT–qPCR. The presented data are expressed as the average ± SEM. In panels (D–I), assays were performed in quadruplicate, and the dashed black and red lines are the results of rB.1.1 and rBA.2 bottom, respectively. Statistically significant differences versus rB.1.1 across timepoints were determined by multiple regression. The FWERs calculated using the Holm method are indicated in the figures.

Our investigations suggested that the increased BA.2 fusogenicity was partly determined by the L371F substitution in the S protein (Fig. 2 and 3). We next aimed to address how BA.2 replication capacity and pathogenicity could be attenuated by mutations in the non-*S* regions. Therefore, we first generated two chimeras using CPER: both were based on ancestral B.1.1 (3), swapping either the upstream or downstream regions of the B.1.1 *S* gene with those of BA.2 (Fig. 4A). We designated these two viruses BA.2up and BA.2down, respectively (Fig. 4A), and intranasally inoculated them, as well as the B.1.1 S-carrying virus (i.e., D614G), into hamsters. We discovered that the body weight loss of BA.2up-infected hamsters was comparable to that of B.1.1-infected ones (Fig. 4B). However, the body weight of BA.2down-infected hamsters was significantly higher than that of B.1.1- and BA.2up-infected ones (Fig. 4B). The viral RNA loads in the oral swabs of these three infection groups were comparable (Fig. 4C). These results suggest that certain mutations downstream of the *S* gene in the BA.2 genome are responsible for attenuating viral pathogenicity in hamsters. To further assess whether the non-*S* region-related mutations in the BA.2 genome affect viral replication capacity, we inoculated B.1.1, BA.2up, and BA.2down into Vero and VeroE6/TMPRSS2 cells. The BA.2down growth kinetics were significantly lower than those of B.1.1 and BA.2up both in Vero (Fig. 4D) and VeroE6/TMPRSS2 cells (Fig. 4E). In summary, these findings suggest that the mutations downstream of the BA.2 *S* gene attenuate viral growth capacity and thus attenuate viral pathogenicity.

Compared to B.1.1, 11 mutations exist downstream of the *S* gene in the BA.2 genome: ORF3a:T223I, E:T9I, M:Q19E, M:A63T, ORF6:D61L, ORF8:S84L, N:P13L, N:ERS31-33del, N:RG203-204KR, N:S413R, and deletions between nucleotides 29,734 and 29,759 in the 3′ untranslated region (3′ UTR; here, we refer to this mutant as “3′UTRdel”) (Fig. 1C). To identify the mutations contributing to the attenuated viral growth capacity, we prepared a series of B.1.1-based recombinant viruses harboring the respective mutations and performed a virus growth assay using Vero and VeroE6/TMPRSS2 cells. However, no single mutants exhibited attenuated growth kinetics (Fig. 4F and G). As multiple mutations could be detected in the BA.2 membrane (M) and nucleocapsid (N) proteins, multiple mutations in either of these viral proteins could potentially synergically act and result in viral growth capacity attenuation. To address this possibility, we generated two additional recombinant viruses possessing all mutations either in the M (M:Q19E/A63T) or N (N:P13L/ERS31-33del/RG203-204KR/S413R) proteins. However, these two recombinant viruses did not attenuate the growth kinetics compared to the parental B.1.1 virus (Fig. 4H and I). In summary, these results suggest that multiple mutations downstream of the *S* gene in the BA.2 genome cooperatively contribute to the attenuated viral growth capacity.

## DISCUSSION

In this study, we revealed that compared to BA.1, BA.2 acquired two traits independently: (i) increasing fusogenicity by the S protein and (ii) attenuating non-S protein-mediated pathogenicity (3). As a result of the former, our molecular phylogenetic analyses suggested that the BA.2 *S* gene might originate from the BA.1 *S* gene. Our *in vitro* cell culture and *in vivo* hamster model-related experiments demonstrated that the L371F substitution in the S protein increased fusogenicity and intrinsic pathogenicity. In addition, we demonstrated that the L371F substitution in the BA.2 S protein is crucial for determining the BA.2 phenotype (such as enhanced fusogenicity) compared to BA.1, by conferring RBD-folding stability. In fact, additional hydrophobic and aromatic amino acid residue substitutions around L371F, the D339H substitution, were observed in the S protein of BA.2.75, a descendant of BA.2 subvariant (24). In the BA.2.75 S RBD, H339 formed a stacking interaction with F371 (24). The RBD stabilization folding around L371F might be related to the Omicron subvariant evolution.

Previous studies revealed that the deletion of certain accessory genes, such as *ORF3a* (25 – 27), *ORF7a* (26, 28), and *ORF8* (26, 29), attenuate viral pathogenicity in experimentally infected animal models. In this study, by creating SARS-CoV-2 mutant lines using reverse genetics, we presented results suggesting that multiple mutations downstream of the *S* gene in the BA.2 genome cooperatively reduce viral growth efficacy *in vitro*, thereby attenuating intrinsic pathogenicity. These data suggest that naturally occurring mutations in the non-*S* region could modulate the intrinsic SARS-CoV-2 pathogenicity.

In this study, we addressed the mutations virologically characterizing BA.2. However, our study also has certain limitations. First, our molecular phylogenetic analyses suggested that the BA.2 *S* could have originated from the BA.1 *S*. However, the bootstrap values of the S protein phylogenetic tree are rather low in various Omicron lineages (Fig. S1B). As previous studies described (30 – 32), this result could be explained by multiple convergent mutations in the *S* gene, particularly in the RBD-encoding region. Another possible explanation could be the low quality of early-sampled Omicron genomes (33). Although we included multiple steps to remove such potential genomes in our phylogenetic analysis (see the Materials and Methods section), contamination of the viral genome sequences with low quality might affect the results. Inferring the S protein phylogeny of the Omicron variants could be essentially challenging.

Second, we presented experimental results suggesting that multiple mutations downstream of the *S* gene in the BA.2 genome attenuate viral growth and intrinsic pathogenicity (Fig. 4). However, we could not determine the crucial mutations for determining this virological phenotype. Related to this, Chen et al. demonstrated that mutations in the *NSP6* gene, upstream of the *S* gene, are key determinants of attenuated Omicron BA.1 pathogenicity (34). The deletion mutation SG3675-3676del in ORF1ab (Fig. 1C; SG106-107del in NSP6) is conserved both in BA.1 and BA.2, and it could thus be screened in our experiments. However, we set out to perform the screening experiments using two chimeric viruses, rBA.2up and rBA.2bottom (Fig. 4A), it might thus be possible that other BA.2-specific mutation(s) upstream of the *S* gene compensated the pathogenicity attenuated by the NSP6 deletion mutation.

Third, we particularly focused on the L371F substitution in the BA.2 S protein that augmented the BA.1 viral infectivity and fusogenicity (Fig. 2). In fact, we demonstrated that the L371F substitution in the BA.1 increased viral growth capacity and pathogenicity (Fig. 3) and contributed to the Sotrovimab resistance (Fig. 2I). Our results strongly suggest that the L371F substitution is a major mutation that characterizes the viral features of the BA.2. As another example, Pastorio et al. described that the R408S substitution in the Wuhan S protein contributes to increased syncytia formation and reduced sensitivity to neutralization by fully BNT162b2-vaccinated sera (35). Moreover, we also observed the potential importance of the R408S substitution on the features of BA.2 S protein, in particular, its pseudovirus infectivity (Fig. 2E and F). The R408S substitution in the BA.2 S protein could thus also be potentially associated with BA.2 S characterization.

In summary, we revealed how the virological phenotypes of SARS-CoV-2 Omicron BA.2 variant could be determined. Over more than 1 year, the prototypic BA.2 was outcompeted by its descendants, such as BA.5 and BA.2.75, and disappeared from the world. However, understanding the molecular mechanisms and selection pressures that led to the emergence of VOCs, even if they have already gone extinct, would be crucial in preparing for the emergence of future variants. In particular, how the mutations in non-S proteins affect viral features remains well-addressed. Further studies investigating the impact of non-S protein mutations would be important in the future.

## MATERIALS AND METHODS

### Cell culture

HEK293 cells (a human embryonic kidney cell line; ATCC, CRL-1573), HEK293T cells (a human embryonic kidney cell line; ATCC, CRL-3216), Lenti-X 293T cells (Takara, Cat# Z2180N), and HOS-ACE2/TMPRSS2 cells (HOS cells stably expressing human ACE2 and TMPRSS2) (36, 37) were maintained in Dulbecco’s modified Eagle’s medium (DMEM) (high glucose) (Sigma-Aldrich, Cat# 6429-500ML) containing 10% fetal bovine serum (FBS, Sigma-Aldrich Cat# 172012-500ML) and 1% penicillin–streptomycin (PS) (Sigma-Aldrich, Cat# P4333-100ML). Vero cells [an African green monkey (*Chlorocebus sabaeus*) kidney cell line; JCRB Cell Bank, JCRB0111] were maintained in Eagle’s minimum essential medium (EMEM) (Sigma-Aldrich, Cat# M4655-500ML) containing 10% FBS and 1% PS. VeroE6/TMPRSS2 cells (VeroE6 cells stably expressing human TMPRSS2; JCRB Cell Bank, JCRB1819) (22) were maintained in DMEM (low glucose) (Wako, Cat# 041-29775) containing 10% FBS, G418 (1 mg/mL; Nacalai Tesque, Cat# G8168-10ML), and 1% PS. Calu-3/DSP_1-7_ cells (Calu-3 cells stably expressing DSP_1-7_) (38) were maintained in EMEM (Wako, Cat# 05608385) containing 20% FBS and 1% PS.

### Viral genome sequence analysis

SARS-CoV-2 genomes and annotation information used in this study were downloaded from the GISAID database (https://www.gisaid.org) as of 26 February 2022 (8,600,684 sequences). We collected 1,688,401 genomes which were (i) isolated from human samples and (ii) annotated as Omicron (including BA.1, BA.1.1, BA.2, and BA.3 lineages). The number of undetermined nucleotides was counted for each genome and 768,483 sequences were selected that (i) have a certain sampling date, (ii) were isolated from humans, (iii) have less than 1,000 undetermined nucleotides in its genome, and (iv) have less than 10 undetermined nucleotides in the S protein region. For the BA.1 and BA.1.1 lineages, 286 genomes were used, which were sampled from 12 August to 30 November 2021. EPI_ISL_10023502 (BA.1) and EPI_ISL_10023526 (BA.1.1), which were both sampled from the Republic of the Congo, are the earliest samples in our data. For BA.2, 35 genomes were used, which were sampled from 24 November 2011 to 10 December 2021. The four genomes sampled in November 2021 were detected in France (EPI_ISL_9796145, 24 November 2021), South Africa (EPI_ISL_8128463 and EPI_ISL_9679276, both 27 November 2021), and India (EPI_ISL_8693579, 28 November 2021). For BA.3, 28 genomes were used, which were sampled from 24 November 2011 to 26 December 2021. All of BA.3 variants sampled during November 2021 (in total 11 genomes) were isolated in South Africa. We also obtained non-Omicron SARS-CoV-2 genomes by (1) two reference genomes [EPI_ISL_402125 (Wuhan-Hu-1, B lineage) and EPI_ISL_406862 (one of the earliest sequences carrying the S D614G mutation, B.1 lineage)]; (2) 20 randomly sampled genomes of each of the B.1.1.318 and B.1.1.519 lineages suggested by Majumdar et al. (39) and Wang et al. (40), respectively; and (iii) randomly sampling five sequences for each month (from January 2021 to August 2021) for every five continents as the study conducted by Viana et al. (13). We excluded genomes that do not have PANGO categories or are assigned as recombinants of different PANGO lineages (i.e., lineage names starting from “X”). To further reduce the impact of recombination in data analysis, we ran a recombination test using RDP4 software v4.101 (41) multiple times. We excluded sequences that are involved in the recombination event, which has less than three sequences reported as recombinants. Finally, 349 Omicron and 275 non-Omicron genomes were used in this study, which were summarized in the following website: https://doi.org/10.55876/gis8.230110hk.

With the 624 SARS-CoV-2 genomes, we generated a multiple alignment using FFT-NS-1 program in MAFFT suite v7.407 (42). Gaps in the multiple alignment were removed referring to the genomic locations in the Wuhan-Hu-1/2019 reference strain. Based on multiple sequence alignments, maximum likelihood-based trees were constructed using IQ-TREE 2 v2.1.3 with -B 1000 -T AUTO options (43). The phylogenetic trees were visualized using ggtree v2.4.1 (44).

### Mutation frequency calculation

Genomic sequences and annotation information of 15,000,410 SARS-CoV-2 used in calculating mutation frequencies were retrieved from the GISAID database (https://www.gisaid.org) (45) on 20 February 2023. Mutation calling with respect to the Wuhan-Hu-1/2019 reference strain was performed by using Nextclade CLI v.2.9.1 (https://clades.nextstrain.org) (46). We filtered out the data of SARS-CoV-2 (i) that was retrieved from a human host; (ii) that was collected before 31 March 2022; (iii) that was from an original passage; (iv) whose nucleotide sequence is longer than 28,000 base pairs; and (v) that contains less than 2% of ambiguous bases. We randomly selected data of 5% of the remaining SARS-CoV-2 of each lineage, resulting in the data of 316, 6,982, and 15,974 SARS-CoV-2 in B.1.1, BA.1, and BA.2 lineages, respectively. The mutation frequency of each lineage was calculated by dividing the number of mutation occurrences by the total number of SARS-CoV-2 in each lineage. The heatmap of mutation frequencies was created using ComplexHeatmap R package v.2.14.0 (47).

### Plasmid construction

Plasmids expressing the codon-optimized SARS-CoV-2 S proteins of B.1.1 (the parental D614G-bearing variant), BA.1, and BA.2 were prepared in our previous studies (2, 3, 48). Plasmids expressing the codon-optimized S proteins of BA.1 S-based derivatives and BA.2 S-based derivatives were generated by site-directed overlap extension PCR using the primers listed in Table S1. The resulting PCR fragment was digested with KpnI (New England Biolabs, Cat# R0142S) and NotI (New England Biolabs, Cat# R1089S) and inserted into the corresponding site of the pCAGGS vector (49). Nucleotide sequences were determined by DNA sequencing services (Eurofins), and the sequence data were analyzed by Sequencher v5.1 software (Gene Codes Corporation). Plasmids for yeast surface display based on pJYDC1 plasmid backbone (Addgene, Cat#162458) were constructed by restriction enzyme-free cloning (50) based incorporation of RBD BA.1 and BA.2 genes (*Saccharomyces cerevisiae* codon usage optimized) purchased from Twist Biosciences (“construct 3”) (51), covering residues 330–528 or site-directed mutagenesis. Primers used for cloning and mutagenesis are listed in Table S1.

### SARS-CoV-2 S-based fusion assay

A SARS-CoV-2 S-based fusion assay (Fig. 2A through D) was performed as previously described (2, 3, 6, 14 – 16, 24, 48). Briefly, on day 1, effector cells (i.e., S-expressing cells) and target cells (Calu-3/DSP_1-7_ cells) were prepared at a density of 0.6–0.8 × 10^6^ cells in a 6-well plate. On day 2, for the preparation of effector cells, HEK293 cells were cotransfected with the S expression plasmids (400 ng) and pDSP_8-11_ (52) (400 ng) using TransIT-LT1 (Takara, Cat# MIR2300). On day 3 (24 hours posttransfection), 16,000 effector cells were detached and reseeded into a 96-well black plate (PerkinElmer, Cat# 6005225), and target cells were reseeded at a density of 1,000,000 cells/2 mL/well in 6-well plates. On day 4 (48 hours posttransfection), target cells were incubated with EnduRen live cell substrate (Promega, Cat# E6481) for 3 hours and then detached, and 32,000 target cells were added to a 96-well plate with effector cells. *Renilla* luciferase activity was measured at the indicated time points using Centro XS3 LB960 (Berthhold Technologies). For measurement of the surface expression level of the S protein, effector cells were stained with rabbit anti-SARS-CoV-2 S S1/S2 polyclonal antibody (Thermo Fisher Scientific, Cat# PA5-112048, 1:100). Normal rabbit IgG (Southern Biotech, Cat# 0111-01, 1:100) was used as a negative control, and allophycocyanin (APC)-conjugated goat anti-rabbit IgG polyclonal antibody (Jackson ImmunoResearch, Cat# 111-136-144, 1:50) was used as a secondary antibody. The surface expression level of S proteins (Fig. 2A and C) was measured using a FACS Canto II (BD Biosciences) and the data were analyzed using FlowJo software v10.7.1 (BD Biosciences). For the calculation of fusion activity, *Renilla* luciferase activity was normalized to the mean fluorescence intensity (MFI) of surface S proteins. The normalized value (i.e., *Renilla* luciferase activity per the surface S MFI) is shown as fusion activity.

### Pseudovirus infection

Pseudovirus infection (Fig. 2E and F; Fig. S2) was performed as previously described (3, 14, 24, 37, 48, 53 – 56). Briefly, lentivirus (HIV-1)-based, luciferase-expressing reporter viruses were pseudotyped with SARS-CoV-2 S proteins. Lenti-X 293T cells (500,000 cells) were cotransfected with 800 ng psPAX2-IN/HiBiT (36), 800 ng pWPI-Luc2 (36), and 400 ng plasmids expressing parental S or its derivatives using TransIT-293 Reagent (Takara, Cat# MIR2700) according to the manufacturer’s protocol. Two days posttransfection, the culture supernatants were harvested and filtrated. The pseudoviruses were stored at –80°C until use. The amount of pseudoviruses prepared was quantified by the HiBiT assay using a Nano Glo HiBiT lytic detection system (Promega, Cat# N3040) as previously described (36, 57). For the measurement of pseudovirus infectivity, the same amount of pseudoviruses (normalized to the HiBiT value, which indicates the amount of HIV-1 p24 antigen) was inoculated into HOS-ACE2/TMPRSS2 cells, and viral infectivity was measured as described above (see the “Neutralization assay” section).

### Western blotting

Western blot (Fig. 2E and F) was performed as previously described (2, 3, 6, 15). For the blot, Lenti-X 293T cells cotransfected with the S expression plasmids and HIV-1-based pseudovirus-producing plasmids (see the “Pseudovirus assay” section above) were used. To quantify the level of the cleaved S2 protein in the cells, equal numbers of cells were washed and lysed in 1× NuPAGE LDS sample buffer (Thermo Fisher Scientific, Cat# NP0007) containing 2% β-mercaptoethanol and incubated at 70°C for 10 min. Then, 10 µL samples were subjected to Western blot. To quantify the level of the S2 protein in the virions, 900 µL culture medium containing the pseudoviruses was layered onto 500 µL 20% sucrose in PBS and centrifuged at 20,000 *g* for 2 hours at 4°C. Pelleted virions were resuspended in 1× NuPAGE LDS sample buffer containing 2% β-mercaptoethanol and incubated at 70°C for 10 min. For protein detection, the following antibodies were used: mouse anti-SARS-CoV-2 S monoclonal antibody (clone 1A9, GeneTex, Cat# GTX632604, 1:5,000), mouse anti-HIV-1 p24 monoclonal antibody (183-H12-5C, obtained from the HIV Reagent Program, NIH, Cat# ARP-3537, 1:5,000), horseradish peroxidase (HRP)-conjugated mouse anti-beta actin (ACTB) monoclonal antibody (clone AC-15, Sigma-Aldrich, Cat# A3854-200UL, 1:50,000), and HRP-conjugated horse anti-mouse IgG antibody (KPL, Cat# 074-1806, 1:10,000). Chemiluminescence was detected using Western BLoT Ultra Sensitive HRP Substrate (Takara, Cat# T7104A) according to the manufacturer’s instructions. Bands were visualized using an iBright FL1500 Imaging System (Thermo Fisher Scientific).

### Yeast surface display

Yeast surface display binding analyses (Fig. 2G) were performed as previously described (17, 24, 51). The *S. cerevisiae* EBY100 yeasts were transformed with pJYDC-RBD plasmids and grown at 30°C overnight (220 rpm, SD-CAA media). For expression, the media 1/9 (58) supplemented with 10 nM DMSO solubilized bilirubin (Sigma-Aldrich, Cat# 14370-1G) was inoculated to starting OD_600_ 0.7–1 by overnight grown culture and cultivated for the following 24 hours at 20°C. The expressed yeast cells were washed in ice-cold PBSB buffer (PBS with 1 mg/mL BSA), aliquoted (100 µL), resuspended in the analysis solution [PBSB buffer, range of concentrations of CF640R succinimidyl ester labeled (Biotium, Cat# 92108) ACE2 peptidase domain (residues 18–740) and 1 nM bilirubin], and incubated overnight. Subsequently, the incubated samples were washed twice with ice-cold PBSB buffer and transferred into a 96-well plate (Thermo Fisher Scientific, Cat# 268200) for automated data acquisition by a CytoFLEX S Flow Cytometer (Beckman Coulter, USA, Cat#. N0-V4-B2-Y4). The APC and fluorescein isothiocyanate (FITC) signals were recorded for 30,000 events per sample. Gating and analysis strategies including the titration curves fitting by nonlinear least-squares regression using Python v3.7 were described previously (51).

### Label-free DSF assay

A label-free DSF assay (Fig. 2H) was performed. RBDs of the amino acid number 322–536 of SARS-CoV-2 variants BA.1, BA.1_L371F, and BA.2 were expressed and purified as previously reported (14) and prepared in PBS buffer at 0.1 mg/mL. Each protein, 10 µL, was added to three grass capillaries. PBS was used as a control. These capillaries were set in Tycho NT.6, and the *T*_*m*_ values of each protein were measured, and the data were analyzed by using the Tycho NT.6 software v1.3.2.880 (59).

### Neutralization assay using Sotrovimab

Neutralization assay (Fig. 2I) was prepared as previously described (3, 14, 24, 37, 53 – 56). Briefly, the SARS-CoV-2 S pseudoviruses (counting ~20,000 relative light units) were incubated with serially diluted Sotrovimab [prepared in our previous study (3)] at 37°C for 1 hour. Pseudoviruses without sera were included as controls. Then, a 40 µL mixture of pseudovirus and serum/antibody was added to HOS-ACE2/TMPRSS2 cells (10,000 cells/50 µL) in a 96-well white plate. At 2 d.p.i., the infected cells were lysed with a Bright-Glo luciferase assay system (Promega, Cat# E2650), and the luminescent signal was measured using a microplate spectrophotometer ARVO X3 (PerkinElmer). The assay of each serum sample was performed in triplicate, and the 50% neutralization titer (NT_50_) was calculated using Prism 9 software v9.1.1 (GraphPad Software).

### SARS-CoV-2 reverse genetics

Recombinant SARS-CoV-2 was generated by CPER as previously described (3, 6, 21, 48). In brief, nine DNA fragments encoding the partial genome of SARS-CoV-2 (strain WK-521, PANGO lineage A; GISAID ID: EPI_ISL_408667) (22) were prepared by PCR using PrimeSTAR GXL DNA polymerase (Takara, Cat# R050A). A linker fragment encoding hepatitis delta virus ribozyme, bovine growth hormone poly A signal, and cytomegalovirus promoter was also prepared by PCR. The corresponding SARS-CoV-2 genomic region and the PCR templates and primers used for this procedure are summarized in Table S1. The 10 obtained DNA fragments were mixed and used for CPER (21). To prepare GFP-expressing replication-competent recombinant SARS-CoV-2, we used fragment 9, in which the *GFP* gene was inserted in the *ORF7a* frame, instead of the authentic F9 fragment (Table S1) (21).

To generate chimeric recombinant SARS-CoV-2 (Fig. 3A and 4A), mutations were inserted in fragment 8 or 9 by site-directed overlap extension PCR or the GENEART site-directed mutagenesis system (Thermo Fisher Scientific, Cat# A13312) according to the manufacturer’s protocol with the primers listed in Table S1. Nucleotide sequences were determined by a DNA sequencing service (Fasmac), and the sequence data were analyzed by Sequencher v5.1 software (Gene Codes Corporation).

To produce chimeric recombinant SARS-CoV-2, the CPER products were transfected into HEK293-C34 cells using TransIT-LT1 (Takara, Cat# MIR2300) according to the manufacturer’s protocol. At 1 day posttransfection, the culture medium was replaced with Dulbecco’s modified Eagle’s medium (high glucose) (Sigma-Aldrich, Cat# R8758-500ML) containing 2% FCS, 1% PS, and doxycycline (1 µg/mL; Takara, Cat# 1311N). At 7 days posttransfection, the culture medium was harvested and centrifuged, and the supernatants were collected as the seed virus. To remove the CPER products (i.e., SARS-CoV-2-related DNA), 1 mL of the seed virus was treated with 2 µL TURBO DNase (Thermo Fisher Scientific, Cat# AM2238) and incubated at 37°C for 1 hour. Complete removal of the CPER products (i.e., SARS-CoV-2-related DNA) from the seed virus was verified by PCR. The working virus stock was prepared from the seed virus as described below (see “SARS-CoV-2 preparation and titration” section below).

### SARS-CoV-2 preparation and titration

The working virus stocks of chimeric recombinant SARS-CoV-2 were prepared and titrated as previously described (2, 3, 5, 6, 12, 14, 15, 24, 48, 60, 61). In brief, 20 µL of the seed virus was inoculated into VeroE6/TMPRSS2 cells (5,000,000 cells in a T-75 flask). One hour postinfection (h.p.i.), the culture medium was replaced with DMEM (low glucose) (Wako, Cat# 041–29775) containing 2% FBS and 1% PS. At 3 d.p.i., the culture medium was harvested and centrifuged, and the supernatants were collected as the working virus stock.

The titer of the prepared working virus was measured as the 50% tissue culture infectious dose (TCID_50_). Briefly, 1 day before infection, VeroE6/TMPRSS2 cells (10,000 cells) were seeded into a 96-well plate. Serially diluted virus stocks were inoculated into the cells and incubated at 37°C for 4 days. The cells were observed under microscopy to judge the cytopathic effect appearance. The value of TCID_50_/mL was calculated with the Reed–Muench method (62).

### Viral genome sequencing

Viral genome sequencing was performed as previously described (14). Briefly, the virus sequences were verified by viral RNA-sequencing analysis. Viral RNA was extracted using a QIAamp viral RNA mini kit (Qiagen, Cat# 52906). The sequencing library employed for total RNA sequencing was prepared using the NEBNext Ultra RNA Library Prep Kit for Illumina (New England Biolabs, Cat# E7530). Paired-end 76-bp sequencing was performed using a MiSeq system (Illumina) with MiSeq reagent kit v3 (Illumina, Cat# MS-102-3001). Sequencing reads were trimmed using fastp v0.21.0 (63) and subsequently mapped to the viral genome sequences of a lineage B isolate (strain Wuhan-Hu-1; GenBank accession number: NC_045512.2) (22) using BWA-MEM v0.7.17 (64). Variant calling, filtering, and annotation were performed using SAMtools v1.9 (65) and snpEff v5.0e (66). Information on the unexpected mutations detected is summarized in Table S2, and the raw data are deposited in DDBJ Sequence Read Archive (accession number: PRJDB15616).

### SARS-CoV-2 infection

One day before infection, Vero cells (10,000 cells) and VeroE6/TMPRSS2 cells (10,000 cells) were seeded into a 96-well plate. SARS-CoV-2 [1,000 TCID_50_ for Vero cells (Fig. 3B, 4D, F, and H); 100 TCID_50_ for VeroE6/TMPRSS2 cells (Fig. 3C, 4E, G, and I)] was inoculated and incubated at 37°C for 1 hour. The infected cells were washed and 180 µL of culture medium was added. The culture supernatant (10 µL) was harvested at the indicated timepoints and used for RT–qPCR to quantify the viral RNA copy number (see the “RT–qPCR” section below). The infection experiments using an airway-on-a-chip system (Fig. 3F and G) were performed as described above (see the “Airway-on-a-chips” section below).

### RT–qPCR

RT–qPCR was performed as previously described (2, 3, 5, 6, 12, 14, 15, 24, 48, 60, 61). Briefly, 5 µL culture supernatant was mixed with 5 µL of 2× RNA lysis buffer [2% Triton X-100 (Nacalai Tesque, Cat# 35501-15), 50 mM KCl, 100 mM Tris-HCl (pH 7.4), 40% glycerol, 0.8 U/µL recombinant RNase inhibitor (Takara, Cat# 2313B)] and incubated at room temperature for 10 min. RNase-free water (90 µL) was added, and the diluted sample (2.5 µL) was used as the template for real-time RT-PCR performed according to the manufacturer’s protocol using One Step TB Green PrimeScript PLUS RT-PCR kit (Takara, Cat# RR096A) and the following primers: forward *N*, 5′-AGC CTC TTC TCG TTC CTC ATC AC-3′; and reverse *N*, 5′-CCG CCA TTG CCA GCC ATT C-3′. The viral RNA copy number was standardized with a SARS-CoV-2 direct detection RT-qPCR kit (Takara, Cat# RC300A). Fluorescent signals were acquired using a QuantStudio 1 Real-Time PCR system (Thermo Fisher Scientific), QuantStudio 3 Real-Time PCR system (Thermo Fisher Scientific), QuantStudio 5 Real-Time PCR system (Thermo Fisher Scientific), StepOne Plus Real-Time PCR system (Thermo Fisher Scientific), CFX Connect Real-Time PCR Detection system (Bio-Rad), Eco Real-Time PCR System (Illumina), qTOWER3 G Real-Time System (Analytik Jena) Thermal Cycler Dice Real Time System III (Takara), or 7500 Real-Time PCR System (Thermo Fisher Scientific).

### Plaque assay

Plaque assay (Fig. 3E) was performed as previously described (2, 3, 6, 14, 15, 24). Briefly, 1 day before infection, VeroE6/TMPRSS2 cells (100,000 cells) were seeded into a 24-well plate and infected with SARS-CoV-2 (1, 10, 100, and 1,000 TCID_50_) at 37°C for 1 hour. A mounting solution containing 3% FBS and 1.5% carboxymethyl cellulose (Wako, Cat# 039-01335) was overlaid, followed by incubation at 37°C. At 3 d.p.i., the culture medium was removed, and the cells were washed with PBS three times and fixed with 4% paraformaldehyde phosphate (Nacalai Tesque, Cat# 09154-85). The fixed cells were washed with tap water, dried, and stained with a staining solution [0.1% methylene blue (Nacalai Tesque, Cat# 22412-14) in water] for 30 min. The stained cells were washed with tap water and dried, and the size of the plaques was measured using Fiji software v2.2.0 (ImageJ).

### Airway-on-a-chips

Airway-on-a-chips (Fig. 3F and G) were prepared as previously described (12, 23, 24). Human lung microvascular endothelial cells (HMVEC-L) were obtained from Lonza (Cat# CC-2527) and cultured with EGM-2-MV medium (Lonza, Cat# CC-3202). For the preparation of the airway-on-a-chip, first, the bottom channel of a polydimethylsiloxane (PDMS) device was precoated with fibronectin (3 µg/mL, Sigma-Aldrich, Cat# F1141). The microfluidic device was generated according to our previous report (67). HMVEC-L cells were suspended at 5,000,000 cells/mL in the EGM2-MV medium. Then, 10 µL of suspension medium was injected into the fibronectin-coated bottom channel of the PDMS device. Then, the PDMS device was turned upside down and incubated. After 1 hour, the device was turned over, and the EGM2-MV medium was added to the bottom channel. After 4 days, airway organoids (AOs) were dissociated and seeded into the top channel. AOs were generated according to our previous report (68). AOs were dissociated into single cells and then suspended at 5,000,000 cells/mL in the AO differentiation medium. Ten microliter suspension medium was injected into the top channel. After 1 hour, the AO differentiation medium was added to the top channel. In the infection experiments, the AO differentiation medium containing either rBA.1 S-GFP, rBA.2 S-GFP, or rBA.1 S:L371F-GFP (500 TCID_50_) was inoculated into the top channel. At 2 h.p.i., the top and bottom channels were washed and cultured with AO differentiation and EGM2-MV medium, respectively. The culture supernatants were collected, and viral RNA was quantified using RT–qPCR (see the “RT–qPCR” section above).

### Microfluidic device

A microfluidic device was generated according to our previous report (24, 67). Briefly, the microfluidic device consisted of two layers of microchannels separated by a semipermeable membrane. The microchannel layers were fabricated from PDMS using a soft lithographic method. PDMS prepolymer (Dow Corning, Cat# SYLGARD 184) at a base-to-curing agent ratio of 10:1 was cast against a mold composed of SU-8 2150 (MicroChem, Cat# SU-8 2150) patterns formed on a silicon wafer. The cross-sectional size of the microchannels was 1 mm in width and 330 µm in height. Access holes were punched through the PDMS using a 6 mm biopsy punch (Kai Corporation, Cat# BP-L60K) to introduce solutions into the microchannels. Two PDMS layers were bonded to a polyethylene terephthalate (PET) membrane containing 3.0 µm pores (Falcon, Cat# 353091) using a thin layer of liquid PDMS prepolymer as the mortar. PDMS prepolymer was spin-coated (4,000 rpm for 60 seconds) onto a glass slide. Subsequently, both the top and bottom channel layers were placed on the glass slide to transfer the thin layer of PDMS prepolymer onto the embossed PDMS surfaces. The membrane was then placed onto the bottom layer and sandwiched with the top layer. The combined layers were left at room temperature for 1 day to remove air bubbles and then placed in an oven at 60°C overnight to cure the PDMS glue. The PDMS devices were sterilized by placing them under UV light for 1 hour before the cell culture.

### Animal experiments

Animal experiments (Fig. 3H, I, 4B, and C) were performed as previously described (2, 3, 6, 12, 14, 15, 24, 60, 61). Syrian hamsters (male, 4-week-old) were purchased from Japan SLC Inc. (Shizuoka, Japan). For the virus infection experiments, hamsters were anesthetized by intramuscular injection of a mixture of 0.15 mg medetomidine hydrochloride/kg of body weight (Domitor, Nippon Zenyaku Kogyo), 2.0 mg midazolam/kg of body weight (Dormicum, Fujifilm Wako, Cat# 135-13791), and 2.5 mg butorphanol/kg of body weight (Vetorphale, Meiji Seika Pharma), or 0.15 mg medetomidine hydrochloride/kg of body weight , 4.0 mg alphaxaone/kg of body weight (Alfaxan, Jurox), and 2.5 mg butorphanol/kg of body weight . SARS-CoV-2 virus (10,000 TCID_50_ in 100 µL) or saline (100 µL) was intranasally inoculated under anesthesia. Left lungs were collected at 5 d.p.i. for RT-qPCR (Fig. 3I). Body weight was recorded daily by 7 d.p.i. Oral swabs were collected at 1, 3, and 5 d.p.i for RT-qPCR (Fig. 4C).

### Fluorescence microscopy

Fluorescence microscopy (Fig. 3D) was performed as previously described (2, 3, 6, 15). Briefly, 1 day before infection, VeroE6/TMPRSS2 cells (10,000 cells) were seeded into 96-well, glass bottom, black plates and infected with SARS-CoV-2 (100 TCID_50_). At 48 h.p.i., GFP fluorescence was observed under an All-in-One Fluorescence Microscope BZ-X800 (Keyence) in living cells, and the 13-square-millimeter-mm^2^ area of each sample was scanned. Images were reconstructed using a BZ-X800 analyzer software (Keyence) and the area of the GFP-positive cells was measured using this software.

### Statistics and reproducibility

Statistical significance was tested using a two-sided Mann–Whitney *U*-test, a two-sided Student’s *t*-test, a two-sided Welch’s *t*-test, or a two-sided paired *t*-test unless otherwise noted. The tests above were performed using Prism 9 software v9.1.1 (GraphPad Software).

In the time-course experiments (Fig. 2B, D, 3B, C, F, and 4B through I), a multiple regression analysis including experimental conditions (i.e., the types of infected viruses) as explanatory variables and timepoints as qualitative control variables was performed to evaluate the difference between experimental conditions thorough all timepoints. The initial time point was removed from the analysis. The *P* value was calculated by a two-sided Wald test. Subsequently, familywise error rates (FWERs) were calculated by the Holm method. These analyses were performed on R v4.1.2 (https://www.r-project.org/).

## Data Availability

The computational codes used in the present study and the GISAID supplemental tables for EPI_SET_230110hk are available in the GitHub repository (https://github.com/ngs923/Omicron_BA.2). The Wuhan-Hu-1 reference genome and working viral stocks are available at the GenBank database (https://www.ncbi.nlm.nih.gov/genbank/) and Sequence Read Archive (accession: PRJDB15616), respectively.

## SUPPLEMENTAL MATERIAL

Fig. S1 jvi.01011-23-s0001.pdf ML trees and genomic mutations of 624 SARS-CoV-2 genomes. The ML trees of 624 SARS-CoV-2 genomes (A), S gene region (B) and RBD-encoding region (C) are shown. In ML trees, a red or blue circle in the internal node is shown if the bootstrap value (1000 times ultrafast bootstrap test) is {greater than or equal to}80% or {greater than or equal to}50%, respectively. In (B), the two numbers in the internal nodes indicate the bootstrap value. In (B) and (C), ML trees are shown in the left, and the mutations of in the corresponding sequence are shown as dot in right.

Fig. S2 jvi.01011-23-s0002.pdf Comparison of viral infectivity pseudotyped with BA.1 S and BA.2 S. HOS-ACE2-TMPRSS2 cells were infected with pseudoviruses bearing each S protein. The amount of input virus was normalized based on the amount of HIV-1 p24 capsid protein. The percent infectivity compared to that of the virus pseudotyped with BA.1 S is shown. Assays were performed in triplicate. The presented data are expressed as the average {plus minus} SD. Each dot indicates the result of an individual replicate. Statistically significant difference (*, *P* < 0.05) versus parental BA.1 was determined by a two-sided Student's *t* test. The red number indicates the fold change between BA.1 and BA.2.

Table S1 jvi.01011-23-s0003.xlsx Primers used in this study.

Table S2 jvi.01011-23-s0004.xlsx Summary of unexpected amino acid mutations detected in the working virus stocks.

ASM does not own the copyrights to Supplemental Material that may be linked to, or accessed through, an article. The authors have granted ASM a non-exclusive, world-wide license to publish the Supplemental Material files. Please contact the corresponding author directly for reuse.
