# Supplementary material for: Multiple mutations of SARS-CoV-2 Omicron BA.2 variant orchestrate its virological characteristics
Source: J Virol. 2023 Oct 5;97(10):e01011-23. doi: 10.1128/jvi.01011-23 (PMC10781145; doi:10.1128/jvi.01011-23)
Supplement: Fig. S1 — ML trees and genomic mutations of 624 SARS-CoV-2 genomes. The ML trees of 624 SARS-CoV-2 genomes (A), S gene region (B) and RBD-encoding region (C) are shown. In ML trees, a red or blue circle in the internal node is shown if the bootstrap value (1000 times ultrafast bootstrap test) is {greater than or equal to}80% or {greater than or equal to}50%, respectively. In (B), the two numbers in the internal nodes indicate the bootstrap value. In (B) and (C), ML trees are shown in the left, and the mutations of in the corresponding sequence are shown as dot in right. [file jvi.01011-23-s0001.pdf]

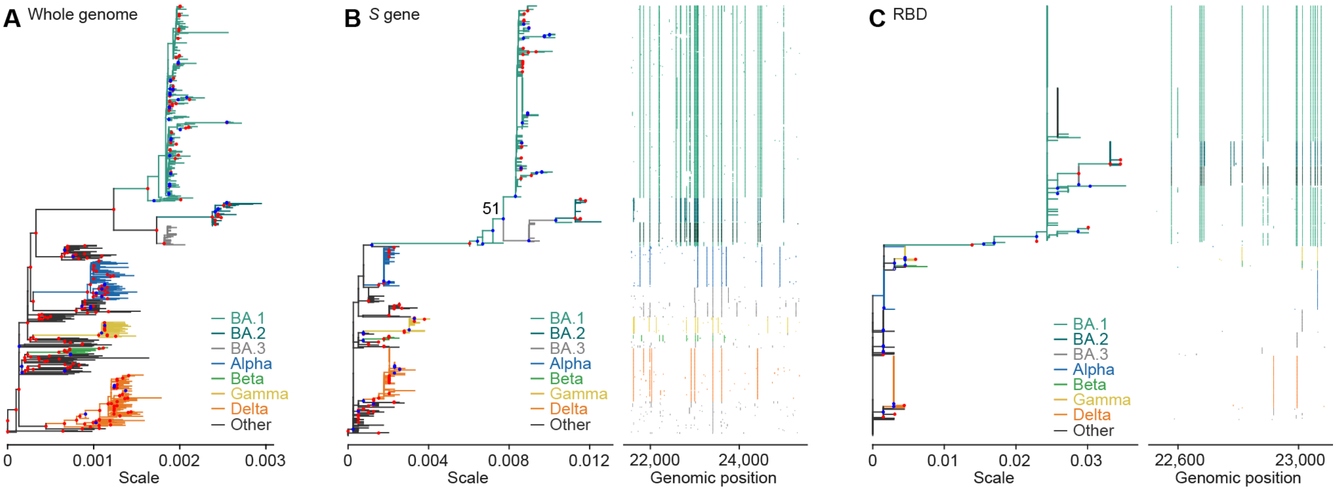

Figure S1. ML trees and genomic mutations of 624 SARS-CoV-2 genomes

The ML trees of 624 SARS-CoV-2 genomes (**A**), *S* gene region (**B**) and RBD-encoding region (**C**) are shown. In ML trees, a red or blue circle in the internal node is shown if the bootstrap value (1000 times ultrafast bootstrap test) is ≥80% or ≥50%, respectively. In (**B**), the two numbers in the internal nodes indicate the bootstrap value. In (**B**) and (**C**), ML trees are shown in the left, and the mutations of in the corresponding sequence are shown as dot in right.
