# Supplementary material for: Multiple mutations of SARS-CoV-2 Omicron BA.2 variant orchestrate its virological characteristics
Source: J Virol. 2023 Oct 5;97(10):e01011-23. doi: 10.1128/jvi.01011-23 (PMC10781145; doi:10.1128/jvi.01011-23)
Supplement: Fig. S2 — Comparison of viral infectivity pseudotyped with BA.1 S and BA.2 S. HOS-ACE2-TMPRSS2 cells were infected with pseudoviruses bearing each S protein. The amount of input virus was normalized based on the amount of HIV-1 p24 capsid protein. The percent infectivity compared to that of the virus pseudotyped with BA.1 S is shown. Assays were performed in triplicate. The presented data are expressed as the average {plus minus} SD. Each dot indicates the result of an individual replicate. Statistically significant difference (*, P < 0.05) versus parental BA.1 was determined by a two-sided Student's t test. The red number indicates the fold change between BA.1 and BA.2. [file jvi.01011-23-s0002.pdf]

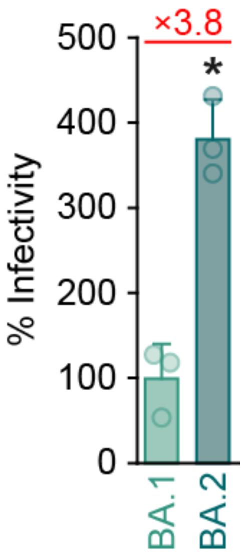

Figure S2. Comparison of viral infectivity pseudotyped with BA.1 S and BA.2 S
HOS-ACE2-TMPRSS2 cells were infected with pseudoviruses bearing each S protein. The amount of input virus was normalized based on the amount of HIV-1 p24 capsid protein. The percent infectivity compared to that of the virus pseudotyped with BA.1 S is shown. Assays were performed in triplicate. The presented data are expressed as the average ± SD. Each dot indicates the result of an individual replicate. Statistically significant difference (*, $P < 0.05$) versus parental BA.1 was determined by a two-sided Student's t test. The red number indicates the fold change between BA.1 and BA.2.
